# Rhabdomyolysis-Induced Acute Renal Failure Following Fenofibrate Therapy: A Case Report and Literature Review

**DOI:** 10.1155/2010/537818

**Published:** 2010-07-25

**Authors:** Ramazan Danis, Sami Akbulut, Sehmus Ozmen, Senay Arikan

**Affiliations:** ^1^Department of Nephrology, Diyarbakir Education and Research Hospital, 21400 Diyarbakir, Turkey; ^2^Department of Surgery, Diyarbakir Education and Research Hospital, 21400 Diyarbakir, Turkey; ^3^Department of Endocrinology and Metabolism, Faculty of Medicine, Dicle Universiy, 21280 Diyarbakir, Turkey

## Abstract

Fenofibrate, a fibric acid derivative, is used to treat diabetic dyslipidemia, hypertriglyceridemia, and combined hyperlipidemia, administered alone or in combination with statins. Rhabdomyolysis is defined as a pathological condition involving skeletal muscle cell damage leading to the release of toxic intracellular material into circulation. Its major causes include muscle compression or overexertion; trauma; ischemia; toxins; cocaine, alcohol, and drug use; metabolic disorders; infections. However, rhabdomyolysis associated with fenofibrate is extremely rare. Herein we report a 45-year-old female patient who was referred to our department because of generalized muscle pain, fatigue, weakness, and oliguria over the preceding 3 weeks. On the basis of the pathogenesis and clinical and laboratory examinations, a diagnosis of acute renal failure secondary to fenofibrate-induced rhabdomyolysis was made. Weekly followups for patients who are administered fenofibrate are the most important way to prevent possible complications.

## 1. Introduction

Rhabdomyolysis is a clinical and biochemical syndrome resulting from skeletal muscle injury and the consequent release of muscle cell constituents into circulation. It may result in myoglobinuria, the filtration of myoglobin into the urine, and is often associated with acute renal failure (ARF) [1]. Its major causes include muscle compression or overexertion; ischemia; toxins; metabolic disorders; cocaine [2], alcohol [2], and drug use; infections [3]. However, drug-induced rhabdomyolysis occurs rarely. In nearly all presented cases, a predisposing factor for rhabdomyolysis, such as high statin dose, diabetes, older age, female gender, renal disease, or hypothyroidism, is present [4, 5]. Herein, we report a 45-year-old diabetic female patient without any known prior renal disease who presented with acute renal failure that developed after fenofibrate treatment.

## 2. Case Report

A 45-year-old female patient presented to our hospital with complaints of muscle pain, weakness, fatigue, decreased urine outflow, and a dark brown urine color for the previous 3 weeks. She had a long history of type 2 diabetes and had had hypertriglyceridemia for about 2 years, which was treated with glimepiride (3 mg daily) and metformin (2 1-g tablets daily). About 3 weeks before presentation, she was prescribed fenofibrate 200 mg daily for hypertriglyceridemia, and she had used it regularly. Her history included diabetic hypertension treated uneventfully with perindopril for 2 years. She had no family history of liver, muscle, or kidney disease, had not traveled recently, and was sexually stable with no history of alcohol, tobacco, or drug abuse. Decreased urine output and generalized weakness (3/5, muscle strength) and muscular tenderness were detected on clinical examination. Laboratory investigations revealed the following levels: serum creatine kinase (CK): 5698 U/L; AST: 179 U/L; ALT: 191 U/L; creatinine: 2.2 mg/dL; urea: 150 mg/dL; LDH: 974 U/L; fasting glucose: 190 mg/dL; HbA1C: 7.85%; triglyceride: 177 mg/dL; total cholesterol: 164 mg/dL; myoglobulin: >100,000 mcg/ml. Her liver and renal functions tests were normal before the fenofibrate therapy. She had no recent viral illness, history of trauma, epilepsy, hypothyroidism, or over-the-counter medication use and had not taken any other medication known to induce rhabdomyolysis. The fenofibrate was discontinued, and intravenous fluid replacement with bicarbonate therapy was started. The myalgia resolved, urine output was normalized, and serum urea and creatinine decreased to normal values on the second day of treatment. The initial, second, and last day laboratory results are presented in Table 1.

## 3. Discussion

Fenofibrate is a derivative of fibric acid. It reduces very low-density lipoprotein (VLDL) and low-density lipoprotein (LDL) and increases high-density lipoprotein (HDL). The side effects of fibrate treatment include gastrointestinal complaints, gallstones, skin reactions, and blood disturbances that are tolerable and reversible. The most important side effect of fenofibrate is rhabdomyolysis [1, 5]. Rhabdomyolysis is a syndrome characterized by muscle necrosis and the release of intracellular muscle contents into systemic circulation. Oliguric or nonoliguric ARF is the most common complication of rhabdomyolysis, occurring in 10%–40% of patients [17]. 

 We established a diagnosis of ARF, which was probably due to fenofibrate-induced rhabdomyolysis based on elevated CK and the absence of any other risk factor related to rhabdomyolysis and ARF according to the Naranjo probability scale [18]. The demographics, medical history, and personal history of this patient did not suggest any underlying disease that may have caused the rhabdomyolysis; however, the time sequence of the start of the fenofibrate and onset of the ARF were consistent with drug-related rhabdomyolysis-related renal injury. Although no rechallenge was attempted, the rapid improvement in muscular enzyme levels and renal function after discontinuation of fenofibrate suggests an association with ARF and rhabdomyolysis related to the use of the drug. Other causes for muscular and/or renal injury (e.g., viruses, toxic exposures, or diabetes mellitus) were possible but not suggested by the clinical history. In our case, rapidly diminishing enzymes on the third day after stopping the fenofibrate therapy indicated that the current clinical situation was associated with fenofibrate.

Acute renal failure secondary to fenofibrate monotherapy-induced rhabdomyolysis is a rare and newly encountered clinical condition, and there are only sixteen cases associated with fenofibrate therapy (nine with monotherapy and seven with fenofibrate-statin combination therapy) which have been published in the English-language literature. Fifteen of them are summarized in Table 2 [1, 4–16]. The risk for serious muscle toxicity appears to be increased in elderly patients and in patients with diabetes [5, 11, 15], renal failure [4, 7, 16], or hypothyroidism [4, 5, 7, 11, 13, 15, 16]. For the patient we present here, tests done 2 months previously found normal results for renal function, thyroid function, and anti-TPO antibody levels. The only risk factor she had was type 2 diabetes mellitus. 

Çetinkaya et al. [5] indicated that dehydration can be an additive risk factor in elderly patients who use fenofibrate; thus, patients in this group should be carefully informed about possible side effects and should be followed closely during the therapy. The patient presented here did not come to any of the controls after starting the therapy, and when she finally came, she had moderate dehydration.

 In conclusion, prescribers and users of fenofibrate should be alert to the possibility of its potentially fatal side effects such as rhabdomyolysis and related ARF. Screening for risk factors such as hepatic impairment, renal insufficiency, serious infections, hypothyroidism, and diabetes should also be considered, especially in elderly patients who are treated with fenofibrate. Doctors who work in regions in which doctor-patient relationships are poor should exercise particular caution when prescribing such drugs.

##  Competing Interests

The authors declare that they have no competing interests.

##  Authors' Contribution

R. Danis, S. Akbulut, and S. Ozmen contributed in writing the paper and review of the literature as well as undertaking a comprehensive literature search; S. Akbulut and S. Arikan contributed to the paper design and preparation.

## Figures and Tables

**Table 1:** Results of our patient's initial, second day, and last day laboratory tests.

Serum	Initial day	Second day	Third day	Reference range
Urea (mg/dL)	29.5	20.3	12.2	10–45
Creatinine (mg/dL)	2.2	1.4	1.1	0.5–1.14
ALT (U/L)	9.7	18.3	31.3	10–35
AST (U/L)	27.6	25.2	24.2	10–40
CK (U/L)	7.8	28.7	19.9	38–174
LDH (U/L)	31.8	23.11	17.10	125–243
Na (mmol/L)	18.5	22.5	19.5	136–145
Ca (mg/dL)	8.0	8.4	8.9	8.4–10.2
K (mmol/L)	3.9	3.6	3.8	3.5–5.1
P (mg/dL)	3.4	2.6	2.3	2.7–4.5

**Table 2:** A summary of reported fifteen cases of rhabdomyolysis associated with fenofibrate therapy.

	References	Year	Age	Sex	Medical History	Other drug	Fenofibrate	BUN	Cr	CK
1	Wu et al. [6]	2009	52	F	DL		200 mg, for 1 mo	43.8		

2	De Souza et al. [7]	2009	54	M	CRF, DL, HT, Hypothyroidism		200 mg, for 2 mo	120	4.9	52749

3	Çetinkaya et al. [5]	2008	60	F	DM-II, HT, DL		200 mg, for NA		4.2	11867

4	Unal et al. [1]	2008	56	F	CAD, DL	Pravastatin	200 mg, for 2 mo	37	2.6	97392
			58	M	CABG, DL	Atorvastatin	200 mg, for 1 mo	36	3.6	96639

5	Yildiz et al. [8]	2008	74	M	CABG, HT, DL		267 mg, for 2 wk	224	5.3	26680

6	Tahmaz et al. [9]	2007	42	F	HT, DL		250 mg, for 4 wk	90	5.5	21000

7	Dedhia and Munsi [10]	2007	68	M	CABGx2, HT, DL	Rosuvastatin	160 mg, for 3 wk		2.3	23665

8	Jacob et al. [11]	2005	70	M	DM-II, HT, DL, Hypothyroidism	Simvastatin	160 mg, for 4 wk		2.7	10936

9	Ireland et al. [12]	2005	67	F	HT, DL	Rosuvastatin	160 mg, for 2 wk	58	3.6	13808

10	Kursat et al. [13]	2005	63	F	HT, DL, Hypothyroidism	Simvastatin	200 mg, for 4 wk	188	4.5	8842

11	Ghosh et al. [14]	2004	58	M	CAD, DL		200 mg, for 5 wk			1129

12	Barker et al. [15]	2003	56	F	HT, DM-II, DL		200 mg, for 10 d		2	5632

13	Clouatre et al. [16]	1999	57	F	CRF, DL, HT, Hypothyroidism		200 mg, for 4 wk			8850
			55	F	CRF, PCRD, DL, Hypothyroidism	Simvastatin	200 mg, for 3 wk			11360

BUN: blood urea nitrogen (mg/dl), Cr: creatinine (mg/dl), DM: diabetes mellitus, CRF: chronic renal failure, CK: serum creatine kinase (IU/L), HT: hypertension, DL: dyslipidemia, CAD: coronary artery disease, CABG: coronary artery by-pass graft, and PCRD: polycystic renal disease.
